# Standardized Patient vs Video Demonstration for Teaching Psychomotor Skills in Spinal Injury Management to Undergraduate Medical Students: Prospective Interventional Comparative Study

**DOI:** 10.2196/78701

**Published:** 2026-06-15

**Authors:** Mondeep Gayan, Sandeep Shrivastava, Kiran Sonowal, Amilee Gogoi, Hrishikesh Goswami, Prasenjit Pathak, Bhawesh Kumar

**Affiliations:** 1 Department of Orthopedics Jorhat Medical College & Hospital Jorhat, Assam India; 2 Department of Orthopedics Datta Meghe Institute of Higher Education & Research (DMIHER) Jawaharlal Nehru Medical College Sawangi (Meghe), Wardha, Maharashtra India; 3 Jawaharlal Nehru Medical College Karnataka Lingayat Education Academy of Higher Education and Research Belagavi, Karnataka India; 4 Department of Orthopedics Lakhimpur Medical College & Hospital North Lakhimpur, Assam India; 5 Department of Pathology Jorhat Medical College & Hospital Jorhat, Assam India; 6 Department of Orthopedics Nagaon Medical College & Hospital Nagaon, Assam India

**Keywords:** psychomotor skills, video demonstration, standardized patient, undergraduate medical education, trauma, emergency

## Abstract

**Background:**

Emergency trauma care in the “golden hour” can reduce the fatality rates by 20%-50%. Imparting trauma care training in the undergraduate Indian medical graduate curriculum can prepare graduates as first responders for both prehospital and hospital setup management. However, teaching these skills for managing acute traumas in a competency-based medical education curriculum is challenging. These skills can be taught on a standardized patient (SP), allowing the learner to achieve higher clinical competency and better communication skills. However, since SP availability is limited, a video demonstration (VD) of such skills is an easy and reliable teaching-learning method.

**Objective:**

This study aims to evaluate the effectiveness of using an SP vs VD for teaching psychomotor skills to undergraduate students in the management of spinal injury.

**Methods:**

A prospective interventional comparative study was performed in the orthopedics department of a teaching hospital from September 2024 to February 2025, with institutional ethics committee approval. Thirty undergraduate students posted in the orthopedics department were divided into 2 groups (15 each) based on their even and odd roll numbers (systematic sampling). One group was shown a VD of the skills like application of an improvised cervical collar and log rolling maneuver for the management of a patient with spinal injury in a primary setting, while the other group was taught on an SP. The students were assessed by an objective structured clinical examination (OSCE), and maneuvers were performed on mannequins. Pretest and posttest evaluations with peer-validated questionnaires along with students’ feedbacks were obtained. The data were analyzed using unpaired 2-sided *t* test for OSCE scores and paired 2-sided *t* test for pretest and posttest scores with statistical significance set at *P*<.05 (95% CI). Student feedback questionnaires based on a 5-point Likert scale were evaluated.

**Results:**

The mean OSCE scores obtained by the VD group (15.47) were significantly different from those obtained by the SP group (18.27) (*P*=.004). However, the mean OSCE scores obtained in individual skill demonstration stations for log rolling maneuver and cervical spine immobilization of the VD group were 2.27 and 3.20 and those of the SP group were 2.33 and 3.27, respectively. This difference was not significant (*P*=.74 and .79, respectively). The student feedback showed that teaching with an SP was marginally more interesting and effective, giving them more confidence to apply these skills in a primary setting.

**Conclusions:**

Both VD and SP methods are equally effective for teaching lifesaving psychomotor skills for the management of spinal injury. However, SP group students had better student engagement. Hence, a blended approach, where VD is used to teach large groups or can be given to students prior to the sessions, followed by teaching or assessing on SPs for application of these skills will lead to optimum results and save time.

## Introduction

Road traffic accidents claim around 1.5 million lives in India every year, contributing to 11% of the total annual global fatalities. Emergency trauma care delivered within the “golden hour”—the critical first hour following major trauma—can reduce the fatality rates by 20%-50%, underscoring the urgent need for rapid intervention [1]. The competency-based medical education (CBME) curriculum gives emphasis on imparting knowledge and basic skills to students to make them competent in patient care [2]. Imparting emergency trauma care training modules in the undergraduate Indian medical graduate curriculum can sensitize students for both prehospital and hospital setup management. As a first responder/bystander, even a trained undergraduate medical student can make a difference and save a life. Teaching lifesaving psychomotor skills for acute trauma management, such as spinal injury care, presents a unique pedagogical challenge, as these skills cannot be practiced on actual patients. Conventional approaches include small-group demonstrations, role play, and video demonstration (VD). Gagliano [3] reported that VD is as good as and often more effective than traditional methods of patient education in increasing short-term knowledge because of role modeling. Ott et al [4] similarly demonstrated that an instructional video, particularly when combined with self-study, serves as an effective standalone tool for promoting the acquisition of practical skills. Padmavathi et al [5] affirmed that VD of skills is an easy and reliable teaching-learning method, as it offers a valuable educational tool for knowledge acquisition, skill development, and attitude changes in health education. However, these skills also include a component in the affective domain, mainly, attitude and communication skills. The Graduate Medical Education Regulation introduces competencies on attitude, ethics, and communication, where communication skills are being taught systematically and phase-wise to Indian medical graduates [6,7]. Standardized patients (SPs) offer a particularly suitable modality for this domain, enabling learners to practice skills on a living, responsive human being in a safe environment, thereby achieving higher levels of clinical competency and more refined communication skills [8]. SP as a teaching modality is widely practiced in simulation-based teaching worldwide and for various health care providers at all levels of learning [9]. However, in limited-income countries, the implementation and evaluation of SP programs remain underexplored, despite their extensive use in high-income nations [10]. Most teaching hospitals in India do not have their own SP training program. The SPs put burden on human resources, finances, and time for proper preparation and implementation. It is also difficult to maintain consistently high-quality learning experiences for students due to SPs’ motivation level, SP training, the available learning resources, and support [11]. There is no conclusive evidence in literature to find out whether VD or SP is a better teaching-learning method in teaching psychomotor skills [12,13]. Yoon et al [14] showed significant benefits in using SPs over video cases in problem-based learning, but the results of their study were based solely on students’ self-reported learning experiences. The authors themselves recommended further research with counterbalanced experimental designs. Direct comparative studies between these two teaching-learning methods remain scarce. This study aims to compare the effectiveness of using SP vs VD for teaching psychomotor skills to undergraduate students in the management of spinal injury in orthopedics.

## Methods

### Study Plan

This prospective interventional study was conducted in the Department of Orthopedics in a teaching tertiary care government hospital in India over a period of 6 months from September 2024 to February 2025. Thirty undergraduate students in this department participated in the study. The undergraduate medical program in India has 3 different phases: phase 1 (preclinical), phase 2 (paraclinical), phase 3 part 1, and phase 3 part 2 (clinical). Those not posted in the orthopedics department, phase 1 students, interns, and absentees were excluded from the study. Systematic sampling (the students were allotted into 2 groups based on their odd and even roll numbers) was done. Data were collected with pretest and posttest questionnaires, objective structured clinical examination (OSCE), the scores attained by the 2 groups, and questionnaires for student feedback. The OSCE checklist and all the questionnaires were certified and validated by peer review by department faculties and members of the college medical education department. This study was performed as an educational research project for 6 months during Advance Course In Medical Education under National Medical Council Nodal Center, JNMC Wardha, under Datta Meghe Institute of Higher Education and Research (DMIHER).

### Ethical Considerations

This study was approved by the institutional ethical committee of Jorhat Medical College & Hospital, Jorhat, Assam, India (EC/NEW/INST/2020/1221) on December 23, 2020 (approval letter number SMEJ/JMCH/MEU/841/Pt III/2023/3809 dated September 2, 2024). All the participants (undergraduate students and ward attendants) who gave their informed voluntary consent to participate in the study were included. Measures were taken to protect the confidentiality and the identity of the participants along with their safety.

### Study Design

A department meeting involving all the faculty members, including postgraduate trainees and staff, was convened to sensitize them about the implementation of the study for undergraduate MBBS students. The teaching material, including videos of demonstrations of such skills, were selected from online YouTube videos of reliable sources or channels (emergency medicine or paramedical training institutes). The videos were evaluated for their content and procedural skills and were peer validated by the faculties in the department and medical education department of the college. Four ward attendants were trained to be SPs of spinal injury, based on the reference from Datta Meghe Institute of Higher Education & Research SP program infused by Mayo Clinic. A spinal injury scenario-based script was drafted in the local language, and the ward attendants were trained extensively and peer validated by department faculties after 2 weeks of training. A mock dry run was done to iron out the practical issues and fine tune the act. The undergraduate students were included in the study after informed consent. Then, the students were divided into 2 groups with systematic sampling. A didactic lecture on Advance Trauma Life Support was taken. One group (even roll numbers) was shown VDs of skills like application of an improvised cervical collar and log rolling maneuver for the management of the patient with spine injury in a primary setting. The other group (odd roll numbers) was taught the same skills on an SP.

The students, under the guidance of the facilitators, were assessed by OSCE for the application of these skills. There were 7 OSCE stations, including 1 rest station: 4 unobserved stations for cognitive assessments and 2 observed stations for psychomotor skill assessments. The students performed the maneuvers on mannequins in the skill laboratory in the observed OSCE stations under 2 independent observers (postgraduate students of orthopedics department) who were blinded (Multimedia Appendix 1). The students were marked as per the preprepared OSCE checklist. Pretest and immediate posttest evaluations were conducted using peer-validated questionnaires in Google forms for all students. Students’ feedbacks were collected on peer-validated questionnaires in Google forms, based on the 5-point Likert scale, to improve the teaching. 

The data so obtained were entered into Microsoft Windows Excel Office (version 2021). The data were compared and evaluated by an unpaired 2-sided *t* test for statistical analysis in case of OSCE scores and paired 2-sided *t* test for pretest and posttest scores, using GraphPad instat 3.0 demo version. A *P* value of less than .05 (95% CI) was considered statistically significant. Student feedback questionnaires based on a 5-point Likert scale were compared. No descriptive statistical analysis of the student feedback was performed.

## Results

**Figure 1:**
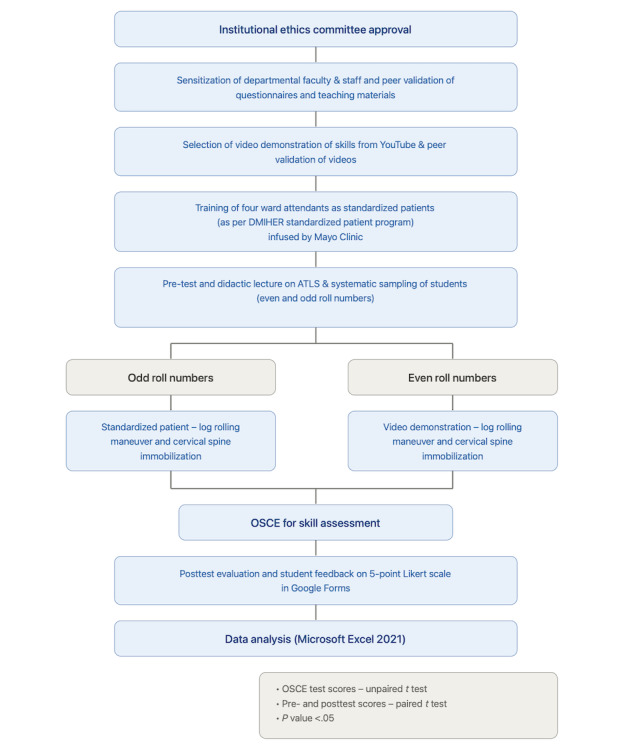
Flowchart of this study. ATLS: Advance Trauma Life Support; DMIHER: Datta Meghe Institute of Higher Education and Research; OSCE: objective structured clinical examination.

Thirty undergraduate students, that is, 8 from phase 2, 14 from phase 3 part 1, and 8 from phase 3 part 2 students gave consent and participated in the study (Figure 1). Following systematic sampling, 15 students were allocated to the VD group and 15 to the SP group, with equal phase-wise distribution across both arms (Table 1).

**Table 1:** Characteristics and results of the study groups stratified by video demonstration and standardized patient conducted in the department of orthopedics in a teaching tertiary care government hospital over a period of 6 months.

Characteristics			Video demonstration		Standardized patient		*P* value^a^
Number of students							
	Total, n	15		15		—^b^	
	Phase 2, n	4		4		—	
	Phase 3 part 1, n	7		7		—	
	Phase 3 part 2, n	4		4		—	
Objective structured clinical examination scores							
	Overall mean (out of 24 marks)	15.47		18.27		.004	
	In log rolling maneuver skill station, mean (out of 4 marks)	2.27		2.33		.74	
	In cervical spine immobilization skill station, mean (out of 4 marks)	3.20		3.27		.79	
Pretest and posttest score							
	Pretest score, mean (out of 7 marks)	3.43		3.50		—	
	Posttest score, mean (out of 7 marks)	5.23		5.53		.35	
	*P* value for difference in pretest and posttest scores	<.001		<.001		—	

^a^*P*<.05 (95% CI) was considered statistically significant.

^b^Not applicable.

**Figure 2:**
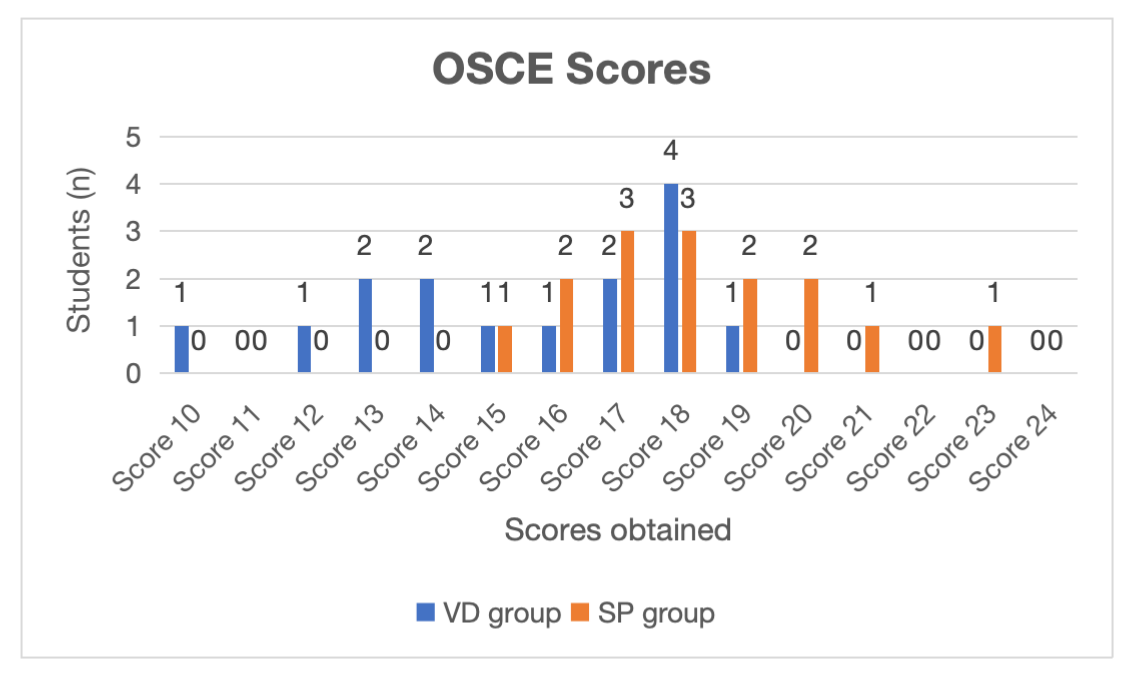
Distribution of the objective structured clinical examination scores (OSCE) in both groups. SP: standardized patient; VD: video demonstration.

The overall OSCE scores across all 30 students ranged from 10 to 23 out of a total of 24 marks, with the most frequently attained score being 18 (achieved by 7 students). When examined by group, the VD group's scores ranged from 10 to 19, with the modal score being 18 (achieved by 4 students). The SP group demonstrated a narrower and higher range, with scores spanning 15 to 23, and a modal score of either 17 or 18 (achieved by 3 students each). The distribution of the OSCE scores across both groups is illustrated in Figure 2. The mean overall OSCE score of the SP group (18.27) was notably higher than that of the VD group (15.47). This difference was statistically significant on unpaired 2-sided *t* test analysis (*P*=.004; Figure 2).

**Figure 3:**
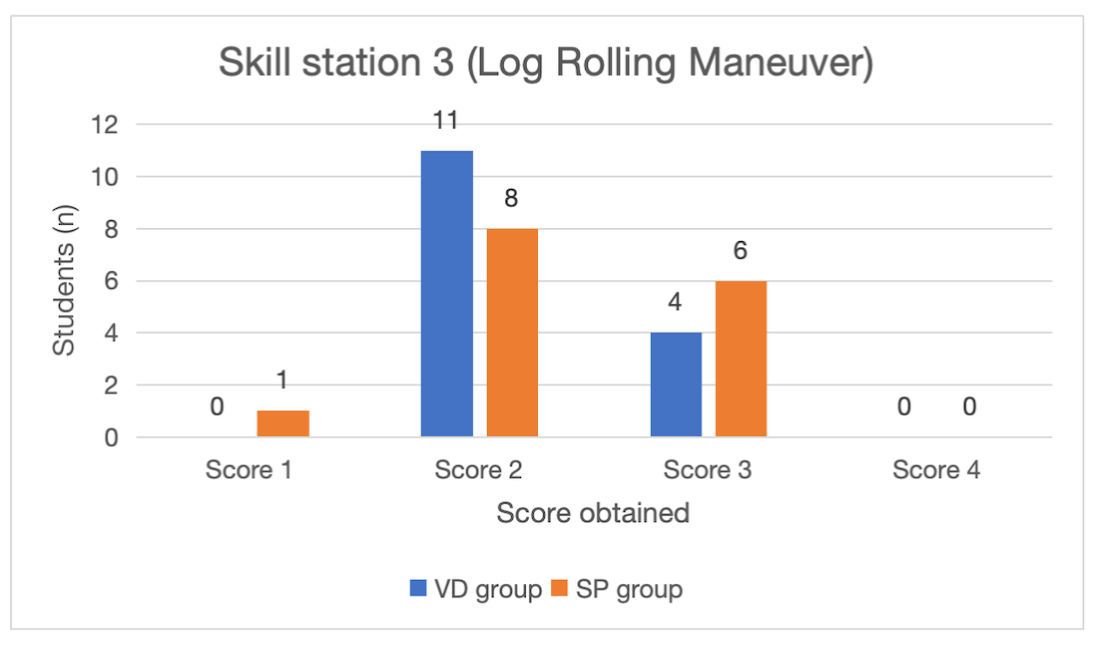
Distribution of the scores in skill station 3 in both groups. SP: standardized patient; VD: video demonstration.

**Figure 4:**
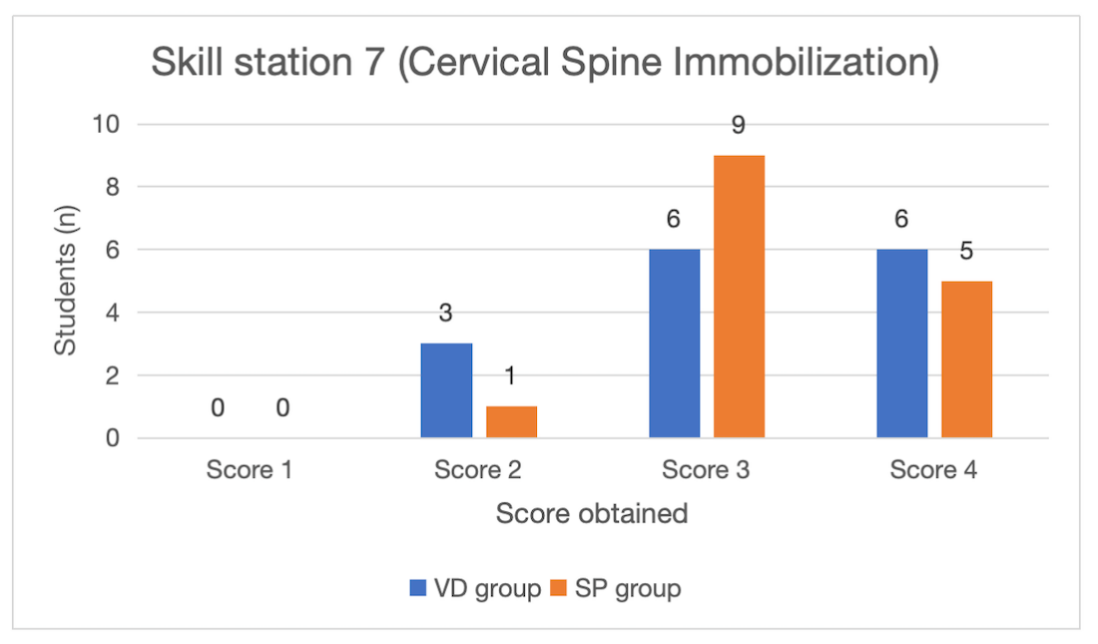
Distribution of the scores in skill station 7 in both groups. SP: standardized patient; VD: video demonstration.

However, when the two observed psychomotor skill stations were analyzed independently, the intergroup differences were no longer statistically significant. For the log-rolling maneuver station (out of 4 marks), the mean scores were 2.27 for the VD group and 2.33 for the SP group (*P*=.74). Similarly, for the cervical spine immobilization station (out of 4 marks), the mean scores were 3.20 and 3.27 for the VD and SP groups, respectively (*P*=.79; Figures 3-4).

For the entire cohort of 30 students, the mean pretest score was 3.47 and the mean posttest score was 5.38 out of a total of 7 marks. This improvement was statistically significant (*P*<.001).

**Figure 5:**
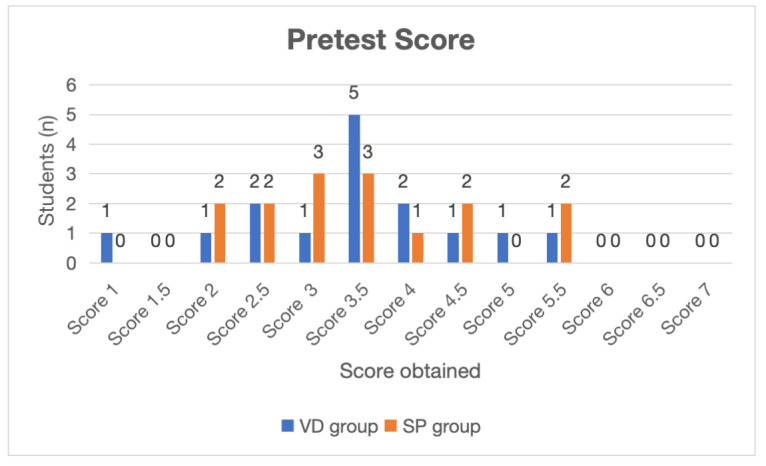
Distribution of the pretest scores in both groups. SP: standardized patient; VD: video demonstration.

Within the VD group, the mean pretest score was 3.43 and the mean posttest score was 5.23, representing a statistically significant improvement (*P*<.001). Similarly, within the SP group, the mean pretest score was 3.50 and the mean posttest score was 5.53, which is also statistically significant (*P*<.001). The distribution of the pretest scores across both groups is shown in Figure 5.

**Figure 6:**
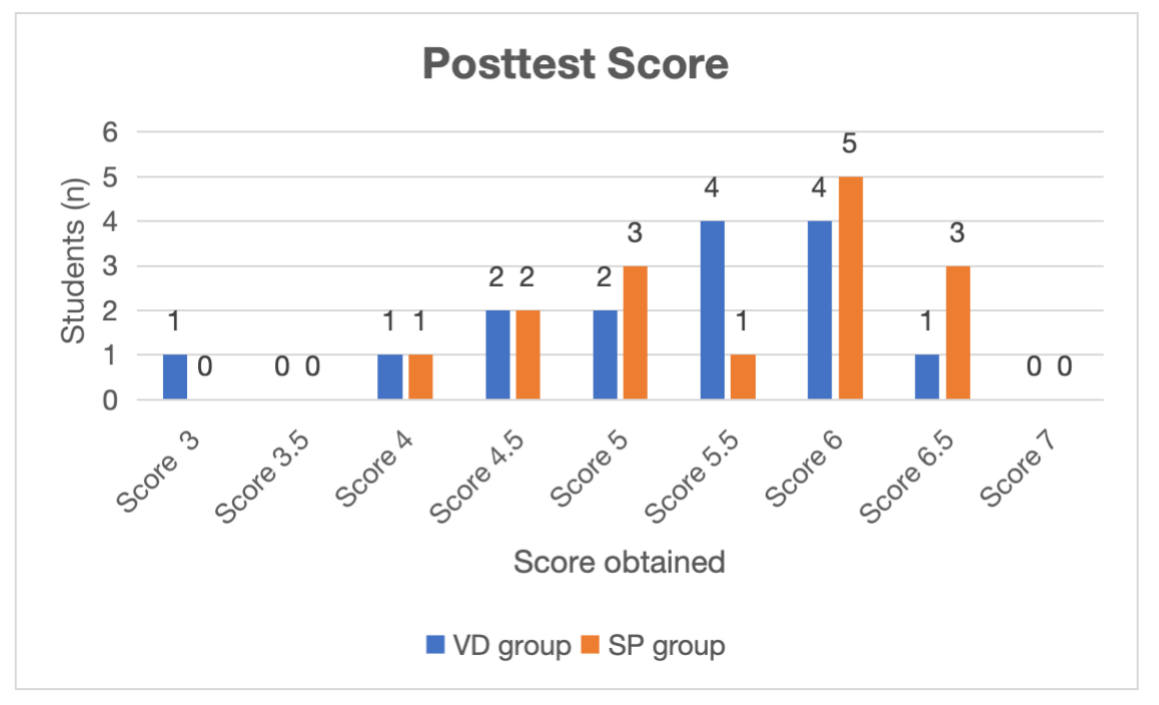
Distribution of the posttest scores in both groups. SP: standardized patient; VD: video demonstration.

The mean posttest score of VD group was 5.23 and that of SP group was 5.53 out of the total of 7 marks. This difference was statistically not significant (*P*=.35) (Figure 6).

**Figure 7:**
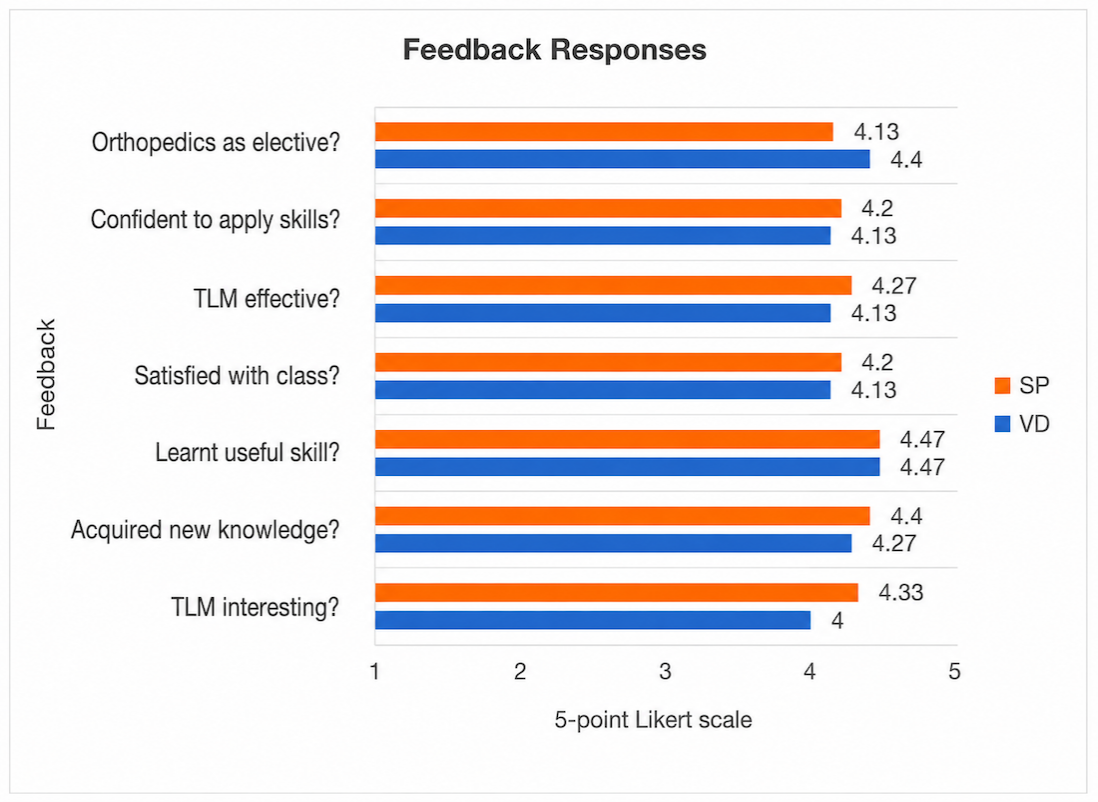
Feedback responses based on the 5-point Likert scale in both groups. TLM: teaching-learning method.

The student feedback was taken in a peer-validated Google form based on 5-point Likert scale regarding the appeal, effectiveness, and outcome of the 2 methods. Overall, students in the SP group rated their learning experience marginally more favorably, finding simulation with an SP to be a more engaging and effective method. Students in the SP group also reported marginally greater satisfaction and confidence in their ability to apply the learned skills in a primary care setting when required (Figure 7).

## Discussion

### Principal Findings

This study compares two teaching-learning methods, namely, VD and SP simulation, for imparting psychomotor skills to undergraduate medical students in the management of acute spinal injury in primary settings. Two groups of 15 students each, drawn from different academic phases, were taught the log-rolling maneuver and cervical spine immobilization technique and subsequently assessed through OSCE and pretest and posttest knowledge evaluations.

The mean OSCE scores obtained by the VD and SP groups were 15.47 and 18.27, respectively. This difference was found to be statistically significant (*P*=.004). However, when the two observed psychomotor skill stations, that is, log-rolling maneuver and cervical spine immobilization, were analyzed independently, no statistically significant intergroup difference was found (*P*=.74 and *P*=.79, respectively), with mean scores of 2.27 vs 2.33 and 3.20 vs 3.27 for the VD and SP groups, respectively. This apparent paradox-significant overall OSCE difference but comparable individual skill station scores warrants reflection. A plausible explanation could be that the students paid more attention to the psychomotor (practical hands-on) aspect of the skill as compared to the cognitive (knowledge) and affective (attitude, communication) parts of the skill, as these sessions were conducted as a part of a study rather something directly related to their examination. It could also be because the OSCE stations were designed more toward testing purely the psychomotor aspect of the skill, as they were designed to be used in primary or prehospital settings. There was a statistically significant improvement in the pretest and posttest scores of both groups (*P*<.001 in VD group and <.001 in SP group). Both teaching-learning methods were equally good methods as compared to traditional methods. However, on comparison of the posttest scores of both groups, this difference in the improvement was statistically not significant (*P*=.35). This parity is likely attributable to the novelty of both methods relative to conventional didactic teaching, which may have equally engaged and motivated students in both arms. This finding aligns with the established understanding that medical simulation complements but does not replace educational activities, which are based on real patient-care experiences [15]. George et al [16] similarly concluded that video teaching was noninferior to bedside teaching within the 10% margin and did not significantly affect the pass/fail or distinction rates. Student feedback, collected on a 5-point Likert scale, indicated a marginal preference for the SP method, with students in that group rating their learning experience as slightly more interesting and effective. They also reported marginally greater satisfaction and confidence in their ability to apply the learned skills in a real primary care setting. Davies et al [17] reported that clinical performance and student self-confidence were positively correlated to the use of SP.

There is no consensus in literature review, as to which teaching-learning method is better for teaching psychomotor skills. Hilal et al [12] concluded that hands-on training is better than VD in teaching surgical skills. They found that hands-on training is superior to VD for teaching vacuum extraction in obstetrics. Yoon et al [14] showed significant benefits in using SPs over video cases in problem-based learning, but they recommended further research with counterbalanced experimental designs. This study responds to this gap by offering a direct assessor-blinded comparison of both modalities by using objective performance measures.

### Strengths

We initiated and developed an SP program in our institute for the first time in spite of limited resources. The 30 students sensitized on trauma care can be force multipliers in disaster management. As a first responder/bystander, even a trained undergraduate medical student can make a difference and save a life, as the skills were designed for a primary prehospital scenario. In our study, we drafted a scenario-based script for SPs as per the skills to be taught, and we trained our department’s 4 ward attendants for 2 weeks and then certified them as SPs for the module. A mock trial run was conducted to test their performance. We provided honorarium payment for their cooperation to keep them motivated. Importantly, these trained SPs have since been utilized beyond the study itself, deployed to train personnel from non–health government departments in emergency trauma care, demonstrating their broader utility as an institutional asset and a potential force multiplier in disaster preparedness.

### Limitations

This study had a small heterogeneous sample size of only 30 students. The students were from all the 3 phases. The phase 3 part 1 and phase 3 part 2 students already have prior exposure and knowledge on the topics and skills under study in the CBME curriculum as compared to phase 2 students. The inclusion of the students in the study was dependent on the student’s clinical posting in orthopedics at that time and their attendance in ward, which might not be the true representation of their respective batches; 7-8 students of each phase are posted in orthopedics in their clinical posting of 2 weeks. The voluntary nature of the study—requiring attendance on 3 separate days for the didactic lecture, skill teaching, allowing time for practice and OSCE assessment—made sustaining participation challenging. Absenteeism, intervening breaks due to examinations, and holidays led us to narrow down to 30 students as a feasible sample size even though we wanted to include more. There were only 6 OSCE stations (total 7 stations, including 1 rest station), where only checklist-based assessment was done. A global rating scale used in conjunction with a detailed checklist for a larger number of OSCE stations may have been a better assessment strategy.

### Conclusions

Both VD of skills and use of SP are equally effective teaching-learning methods for teaching psychomotor skills, which are difficult to teach on actual emergency patients in acute scenarios. However, the SP group had better overall OSCE scores and better student engagement. Both VD and SP methods, when used in conjunction, can give optimum results saving on time and resources rather than when used in isolation. The training and development of SPs for teaching skills is time-, finance-, and human resource–intensive, but it can give students a real life-like immersive learning experience and make them more confident in the application of the acquired skills in real patients. A good quality standard VD of skills provides both audiovisual input to students for role modelling and is equally visible and audible to everyone. A blended approach is therefore recommended: high-quality VD can be used for large-group instruction or as presession preparatory material, following which students are taught and assessed on SPs for skill application and consolidation. This sequential model harnesses the scalability and consistency of video-based learning alongside the experiential depth and interactivity of SP simulation.

### Implications

As per government policy, more medical colleges are being opened and a greater number of students are being enrolled to increase the number of doctors per population in India. There is paucity of trained faculties and adequate number of patients to teach undergraduate students. As per the CBME curriculum, which gives more emphasis on competency and skills, we would like to recommend a blended approach of mix and match of technology—novel methods along with the traditional method is the way forward. More such detailed studies with large sample sizes and of longer duration should be performed on blending of teaching-learning methods.

## Data Availability

The datasets generated or analyzed during this study are available from the corresponding author on reasonable request.

## Appendix

Multimedia Appendix 1 Photographs of the sessions conducted for the objective structured clinical examination.

## Abbreviations

CBME: competency-based medical education
OSCE: objective structured clinical examination
SP: standardized patient
VD: video demonstration
