# Supplementary material for: Standardized Patient vs Video Demonstration for Teaching Psychomotor Skills in Spinal Injury Management to Undergraduate Medical Students: Prospective Interventional Comparative Study
Source: JMIR Form Res. 2026 Jun 15;10:e78701. doi: 10.2196/78701 (PMC13316020; doi:10.2196/78701)
Supplement: Multimedia Appendix 1 [file formative_v10i1e78701_app1.docx]

**APPENDIX**


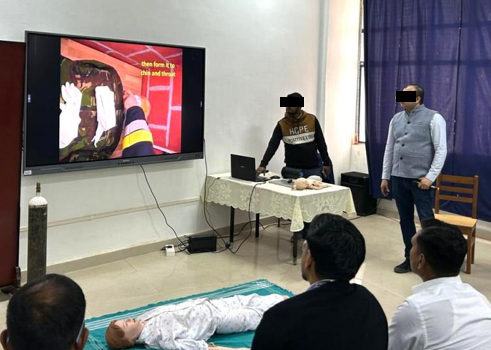


1. Cervical spine immobilization video


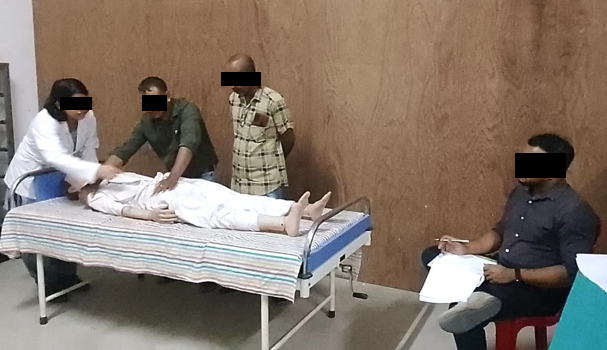


1. OSCE Skill station 3 log rolling


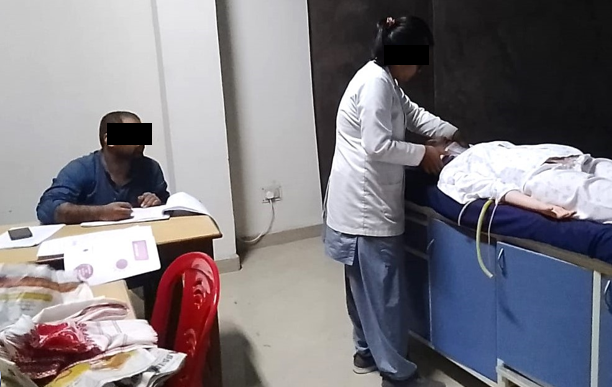


1. OSCE skill station 7 cervical spine immobilization
